# Synergistic Encapsulation of Paclitaxel and Sorafenib by Methoxy Poly(Ethylene Glycol)-*b*-Poly(Caprolactone) Polymeric Micelles for Ovarian Cancer Therapy

**DOI:** 10.3390/pharmaceutics15041206

**Published:** 2023-04-10

**Authors:** Chae Eun Jin, Moon Sup Yoon, Min Jeong Jo, Seo Yeon Kim, Jae Min Lee, Su Jeong Kang, Chun-Woong Park, Jin-Seok Kim, Dae Hwan Shin

**Affiliations:** 1 College of Pharmacy, Chungbuk National University, Cheongju 28160, Republic of Korea; cejin@chungbuk.ac.kr (C.E.J.);; 2 Drug Information Research Institute (DIRI), College of Pharmacy, Sookmyung Women’s University, Cheongpa-ro 47-gil 100, Yongsan-gu, Seoul 04310, Republic of Korea

**Keywords:** ovarian cancer, paclitaxel, sorafenib, combination therapy, mPEG-*b*-PCL, micelle, antitumor, pharmacokinetics

## Abstract

Ovarian cancer has a high mortality rate due to difficult detection at an early stage. It is necessary to develop a novel anticancer treatment that demonstrates improved efficacy while reducing toxicity. Here, using the freeze-drying method, micelles encapsulating paclitaxel (PTX) and sorafenib (SRF) with various polymers were prepared, and the optimal polymer (mPEG-*b*-PCL) was selected by measuring drug loading (%), encapsulation efficiency (%), particle size, polydispersity index, and zeta potential. The final formulation was selected based on a molar ratio (PTX:SRF = 1:2.3) with synergistic effects on two ovarian cancer cell lines (SKOV3-red-fluc, HeyA8). In the in vitro release assay, PTX/SRF micelles showed a slower release than PTX and SRF single micelles. In pharmacokinetic evaluation, PTX/SRF micelles showed improved bioavailability compared to PTX/SRF solution. In in vivo toxicity assays, no significant differences were observed in body weight between the micellar formulation and the control group. The anticancer effect of PTX/SRF combination therapy was improved compared to the use of a single drug. In the xenografted BALB/c mouse model, the tumor growth inhibition rate of PTX/SRF micelles was 90.44%. Accordingly, PTX/SRF micelles showed improved anticancer effects compared to single-drug therapy in ovarian cancer (SKOV3-red-fluc).

## 1. Introduction

Ovarian cancer (OC) can be fatal as it penetrates deep into the organs and is difficult to detect at an early stage [1]. More than 70% of ovarian cancers are stage three or higher at the time of diagnosis; therefore, the prognosis after treatment is poor. In the case of stage one ovarian cancer, a cure rate of more than 90% can be achieved with only surgical resection; however, the frequency of stage one OC is less than 10% of the total [2]. Over 70% of patients who are diagnosed with stage three or higher OC experience a recurrence, and stage three to four OC is a more lethal cancer, with a 5-year survival rate of only 17–39% [3].

The standard treatment for OC is surgical removal and staging [4,5]. Even in the case of stage one OC, chemotherapy is administered concurrently when it has higher grade [6]. Paclitaxel (PTX) is a standard-of-care anticancer drug for treating OC and is used in combination with carboplatin [7]. The mechanism of action of PTX is to stabilize the polymerization state of microtubules and inhibit their degradation into monomeric tubulin [8]. In addition, PTX can inhibit tumor growth by modulating several interactions in the tumor microenvironment (TME) [9]. Consequently, this inhibits spindle formation, which is necessary for cell division, thereby inhibiting cancer cell division and growth. 

Although PTX is known to exhibit excellent anticancer effects, OC cells easily acquire PTX resistance [10,11]. When resistance to PTX is achieved, disease treatment becomes difficult and the recurrence rate increases. Cancer stem cells (CSCs) contribute to OC resistance [12,13]. CSCs can induce chemoresistance because they are capable of self-renewal and self-differentiation [14]. Chemical resistance has been overcome by combination therapy [15,16,17]. Sorafenib (SRF), which will be used as a combination therapy with PTX for OC in this study, is one of the targeted anticancer drugs of the tyrosine kinase inhibitor (TKI) class [18]. SRF exerts anticancer effects by targeting multiple receptor tyrosine kinases and inhibiting the signal transduction system in cancer cells [19,20]. In addition, SRF inhibits the functions of platelet-derived growth factor receptor (PDGFR) and Vascular Endothelial Growth Factor Receptor (VEGFR) to prevent cancer cell differentiation or angiogenesis [20,21]. The combination of PTX and SRF selected to overcome chemoresistance can effectively target cancer stem cells [22,23]. The anti-tumor evaluation of the miPS-BT549cmP and miPS-Huh7cmP cell lines, which are CSCs derived from mouse-induced pluripotent stem cells, confirmed that the combination of PTX and SRF exhibits a synergistic effect [22].

Combination therapy with PTX and SRF, which have different mechanisms of action, is expected to generate synergy by targeting CSCs [22,24,25]. As such, PTX and SRF have the potential for combination therapy in the treatment of OC. However, due to their low solubility in water, they present a problem when used as intravenous combination therapy [26]. The existing formulations have attempted to solve the problem of poor solubility of PTX by using a solubilizer (Cremophor^®^); however, this caused a toxicity issue [27]. In addition, SRF, which is generally a poorly soluble drug, is administered orally because intravenous administration is difficult [28]. When comparing intravenous administration of PTX using a solubilizer and oral administration of SRF to treat OC, the disadvantages due to overlapping toxicity outweigh their advantages [29]. Previous studies have shown that the disadvantages of toxicity outweigh the advantages of the oral administration of intravenous PTX and SRF [20]. To maximize the therapeutic effect of SRF and PTX combination therapy injection, a toxicity-reducing solution must be used, such as nanoparticles [30,31].

Therefore, in this study, we prepared micelles encapsulated with PTX/SRF to increase drug solubility and lower toxicity. It has been reported that the micelles made of the polymer (mPEG-*b*-PCL) are biocompatible and biodegradable [20,32]. In addition to their low toxicity, micelles, which are nano-pharmaceuticals, can increase bioavailability. Micelles have the advantage of surviving longer in vivo because their hydrophilic shell prevents the phagocytosis of macrophages [33]. Micelles encapsulated with intravenously administered anticancer drugs must remain in circulation for a long time and accumulate in the tumor tissue before being cleared by the reticuloendothelial system [34]. Therefore, it must be effectively internalized within cancer cells. Additionally, the surface charge of micelles can affect the stability of the formulation and its interaction with cells. The absolute value of the surface charge is the most significant factor when micelle formation is eliminated by phagocytosis [35]. Consequently, smaller absolute values of zeta potential will prevent macrophage uptake more effectively. In the case of micelles with a negative surface charge, the absorption rate of phagocytes is low; therefore, the drug can remain in vivo longer [33]. Micelles, in particular, have the characteristic of retaining the drug in the tissues around cancer cells for a prolonged period due to the enhanced permeability and retention (EPR) effect [36,37].

In this study, micelles were prepared with mPEG-*b*-PLA, F-127, Soluplus^®^, and mPEG-*b*-PCL. Micelles made with Soluplus^®^ can improve tumor sensitivity in vivo by inhibiting P-glycoprotein-mediated drug efflux [38]. Nanoparticles made of F-127 promote effective drug transport and sustained drug delivery [39]. mPEG-*b*-PLA can improve the solubility and pharmacokinetic profile of encapsulated drugs when made into micelles [40]. As such, micellar formulations have the advantage of effectively delivering poorly soluble drugs to target cancers.

In this study, mPEG-*b*-PCL, an amphiphilic polymer with biodegradability, biocompatibility, and biostability, was used [41]. Subsequently, PTX and SRF were simultaneously encapsulated in micelles to examine their synergistic effects on human OC cell lines. The bioavailability, toxicity, and tumor growth inhibitory ability of the final formulation were evaluated through in vivo analysis using experimental animals. In a previous study, anticancer evaluation was conducted in liver or breast cancer for nanoparticles encapsulated with PTX/SRF, and the possibility was proven [42,43]. The novelty of this study lies in the use of mPEG-*b*-PCL micelles encapsulated with PTX/SRF evaluated in OC.

## 2. Materials and Methods

### 2.1. Materials and Reagents

Soluplus^®^ (polyvinyl caprolactam-polyvinyl acetate-polyethylene glycol graft copolymer; PCL-PVAc-PEG) was donated by BASF (Ludwigshafen, Rhineland-Palatinate, Germany). Methoxy poly (ethylene glycol)-*b*-poly (D, L-lactide) (mPEG[4000]-*b*-PLA[2200]) was purchased from Advanced Polymer Materials Inc. (Montreal, QC, Canada). Poly (ethylene oxide-*b-ε*-caprolactone) (PEO[2000]-*b*-PCL was from Polyscitech^®^ (West Lafayette, IN, USA), and Pluronic^®^ F-127 was purchased from Sigma-Aldrich Corp. (St. Louis, MO, USA). Buffered saline (DPBS), trypsin, Cremophor EL^®^, Matrigel, D–luciferin potassium salt (GoldBio, St. Louis, MO, USA), and Roswell Park Memorial Institute medium (RPMI 1640) were purchased from Corning Inc. (New York, NY, USA). PTX, SRF, and genistein were purchased from LC Laboratories (Woburn, MA, USA). Methanol was purchased from Honeywell Burdick & Jackson (Ulsan, Republic of Korea), and ethanol and acetonitrile (ACN) were purchased from Fisher Scientific Ltd (Waltham, MA, USA). Distilled water was purchased from Tedia (Fairfield, OH, USA) and Tert-Butanol was from Sigma-Aldrich Corp. (St. Louis, MO, USA). All solvents for high-performance liquid chromatography (HPLC) were HPLC grade or higher.

### 2.2. Cell Lines and Culture Conditions

The human OC cell line HeyA8 was gifted by Dr. Jeong-won Lee at Seoul Samsung Hospital. SKOV3-red-fluc, a cancer cell line in which a fluorescent factor was introduced into SKOV3, a human OC cell line, was purchased from PerkinElmer (Waltham, MA, USA). All cell lines were grown in Roswell Park Memorial Institute medium (RPMI 1640) supplemented with streptomycin/penicillin and 10% (*v*/*v*) fetal bovine serum. Cells were cultured in a 5% CO_2_, 37 °C environment.

### 2.3. Combination Index (CI) Analysis

The Combination Index (CI) value is a concept that serves as a basis for judging the synergistic effect between concomitant drugs. CI analysis was performed using Chou–Talalay’s method [44].

$$\mathrm{Combination}\;\mathrm{Index}\;\left(\mathrm{CI}\right)=\frac{{\left(D\right)}_{1}}{{\left(D_{x}\right)}_{1}}+\frac{{\left(D\right)}_{2}}{{\left(D_{x}\right)}_{2}}$$

D_1_ and D_2_ are the IC_50_ values obtained by considering the mole fraction of the IC_50_ (50% inhibitory concentration) value of each combination drug, and D_x1_ and D_x2_ are the IC_50_ values of a single drug. CI < 1, = 1, and > 1 indicate synergism, additive effect, and antagonism, respectively. In addition, CI values of 0.1–0.3 are subdivided into strong synergism, 0.3–0.7 synergism, 0.7–0.85 moderate synergism, and 0.85–0.90 weak synergism [45,46,47].

### 2.4. HPLC Analysis

An HPLC system was used for the concentration analysis of PTX and SRF. All samples were obtained from in vitro and in vivo assays throughout this study. The HPLC system consisted of a Waters 2695 separation module and Waters 2996 photodiode array detector (Waters, Milford, MA, USA). A Fortis C18 chromatography column (5 μm, 4.6 × 250 mm) (Fortis Technologies Ltd., Cheshire, UK) was maintained at 30 °C. PTX, SRF, and genistein (internal standard, IS) were eluted in isocratic mode. The mobile phase composition was ACN:water = 7:3 (*v*/*v*) and the flow rate was 1.0 mL/min. The retention times of PTX, SRF, and IS were 5.4, 8.8, and 3.4 min, respectively. PTX, SRF, and IS were detected at 227.4 nm, 264.0 nm, and 259.3 nm, respectively. Concentrations were calculated using a calibration curve and quantified by substituting unknown sample values into the calibration curve.

### 2.5. Formation of the PTX/SRF-Encapsulated Polymeric Micelles

**Figure 1:**
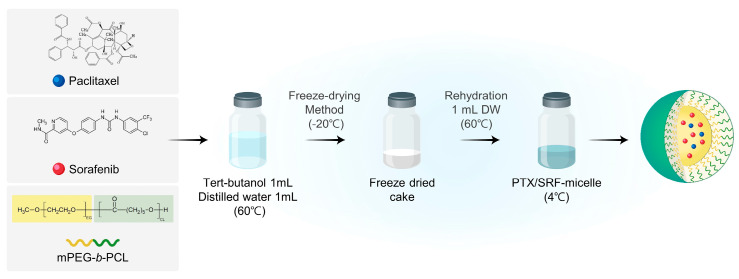
Schematic diagram of the preparation process for paclitaxel (PTX)- and sorafenib (SRF)-encapsulated mPEG-*b*-PCL micelles (PTX/SRF micelles) using the freeze-drying method.

PTX-, SRF-, and PTX/SRF-encapsulated micelles were produced through lyophilization (Figure 1). First, 3 mg of PTX, 3 mg of SRF, and 60 mg of polymer were weighed in a scintillation vial. Following this, 1 mL of tert-butanol preheated to 60 °C was added and sonicated for 10 min. When the drug was completely dissolved in the solvent and became transparent, 1 mL of sterilized distilled water was added, heated to 60 °C, and shaken in a 60 °C water bath. Vortexing was repeated five times. Afterward, it was rapidly frozen for 1 h in a –70 °C deep freezer. The frozen sample was placed in a lyophilizer (Advantage Pro; SP Scientific, Warminster, PA, USA) and freeze-dried for 24 h at –20 °C and 500 mTorr. For hydration, 1 mL of sterile distilled water heated to 60 °C was added, followed by vortexing and sonication. The hydrated micelles were transferred to an EP tube and centrifuged at 4 °C and 16,600× *g*. for 5 min in a centrifuge (Hanil Science Inc., Gimpo, Republic of Korea) to remove excess polymer and unencapsulated drugs from the supernatant. PTX/SRF-encapsulated mPEG-*b*-PCL micelles were produced by filtering with a 1 mL syringe and a 200 nm filter (Corning, New York, NY, USA).

### 2.6. Physicochemical Micelle Characterization

The particle size, polydispersity index (PDI), and zeta potential of PTX/SRF micelles were measured using a dynamic light scattering (DLS) instrument (Anton Paar, Litesizer 500, Graz, Austria). The particle size angle was automatically selected by the program between the side scatter (90°) and backscatter (175°). HPLC was used to quantify the amount of drug contained within the PTX/SRF micelle, and the encapsulation efficiency (EE, %) and drug loading (DL, %) were calculated.

$$\mathrm{EE}\;\left(\%\right)=\left(\frac{\mathrm{weight}\;\mathrm{of}\;\mathrm{drug}\;\mathrm{in}\;\mathrm{micelles}}{\mathrm{weight}\;\mathrm{of}\;\mathrm{initial}\;\mathrm{feeding}\;\mathrm{drug}}\right)\times 100$$

$$\mathrm{DL}\;\left(\%\right)=\left(\frac{\mathrm{weight}\;\mathrm{of}\;\mathrm{drug}\;\mathrm{in}\;\mathrm{micelles}}{\mathrm{weight}\;\mathrm{of}\;\mathrm{initial}\;\mathrm{feeding}\;\mathrm{drug}\;\mathrm{and}\;\mathrm{polymer}}\right)\times 100$$

### 2.7. Transmission Electron Microscopy Observation

Micelle images were obtained using a transmission electron microscope (TEM; JEM-2100 Plus, JEOL, Tokyo, Japan). The PTX/SRF micelles prepared with mPEG-*b*-PCL were sampled after diluting them 50-times with sterile distilled water (final concentration of PTX: 56.6 ± 2.48 µg/mL and SRF: 55.0 ± 3.18 µg/mL). The diluted micellar suspension was dropped on the center of a Formvar/Carbon 200 Mesh Cu lattice and then air-dried and fixed at room temperature for 72 h without additional treatment. Images of the PTX/SRF micelles were obtained using a transmission electron microscope at 200 kV.

### 2.8. In Vitro Stability Test

The final formulation of PTX/SRF micelles (stock concentration is 2.83 ± 0.124 mg/mL and 2.75 ± 0.159 mg/mL in the order of PTX and SRF) was evaluated at a refrigerated storage temperature (4 °C) to determine their storage stability. Each sample was diluted 20-fold in sterile distilled water on days 0, 1, 2, 3, 5, 7, 10, and 14, and stock samples were measured immediately on a dynamic light scattering (DLS) instrument. Particle size, polydispersity index (PDI), and zeta potential were measured.

### 2.9. In Vitro Drug Release Assay

Dialysis using phosphate-buffered saline (PBS, pH 7.4) was used to investigate the in vitro drug release pattern. After adding 2 L of PBS to a beaker, 1 mL of each sample (PTX-micelles, SRF micelles, and PTX/SRF micelles) was injected into a dialysis bag (molecular weight cutoff = 20 kDa). The volumes of samples and media were determined considering sink conditions. The sink conditions refer to a state in which the amount of drug released from the dosage form is immediately and infinitely diluted in the surrounding medium. In in vitro drug release studies, sink conditions that are preferred are biorelevant [48]. After sealing the end to prevent leakage, it floated with a buoy and was stirred at 200 rpm at 37 °C. The pre-warmed PBS medium was periodically changed after 8, 24, 72, 168, and 240 h [49]. Sample collections of 20 μL were obtained at 0, 2, 4, 6, 8, 24, 48, 72, 168, 240, and 336 h. The obtained sample was diluted 10-fold with 180 μL of ACN, and HPLC was used to quantitatively analyze the concentrations of PTX and SRF.

### 2.10. In Vitro Cytotoxicity Assay

The toxicity of PTX and SRF on OC cells was investigated using a thiazolyl blue tetrazolium bromide (MTT) assay. HeyA8 (4 × 10^3^ cells/well) and SKOV3-red-fluc (1 × 10^4^ cells/well) cells were seeded in 96-well plates. After 24 h, they were treated with free PTX, free SRF, free PTX/SRF, PTX micelles, SRF micelles, and PTX/SRF micelles. For free drug, the poorly soluble drug was dissolved in DMSO and diluted 1000-times with the medium to obtain the starting concentration. Additionally, the micelles were directly diluted with the medium PTX micelles, PTX/SRF micelles were diluted 1000-times and SRF micelles were diluted 100-times (n = 6). After 48 h of drug treatment, the medium was removed, and 100 μL/well of MTT reagent was added and cultured. After 4 h, the MTT reagent was removed, and a color reaction was induced by adding DMSO in the dark. After stirring for 10 min, cell viability was evaluated by measuring the absorbance at 540 nm using a microplate reader (Spectra Max ID3, Molecular Devices, San Jose, CA, USA). Data processing was performed using GraphPad Prism v. 8.0 (GraphPad Software, La Jolla, CA, USA).

$$\mathrm{Cell}\;\mathrm{viability}\;\left(\%\right)=\left(\frac{\mathrm{The}\;\mathrm{absorbance}\;\mathrm{of}\;\mathrm{the}\;\mathrm{experimental}\;\mathrm{group}}{\mathrm{The}\;\mathrm{absorbance}\;\mathrm{of}\;\mathrm{the}\;\mathrm{control}\;\mathrm{group}}\right)\times 100$$

### 2.11. In Vitro Clonogenic Assay

SKOV3-red-fluc cells (200 cells/well) were seeded in 6-well plates. We treated the free PTX, free SRF, free PTX/SRF, PTX micelles, SRF micelles, and PTX/SRF micelles after 24 h. After 2 weeks of culture, the medium was removed; 0.5% *w*/*v* crystal violet 1 mL/well was added and incubated for 30 min. The stained colonies were gently washed with water and dried to count the number of colonies [50].

$$\mathrm{Colony}\;\mathrm{formation}\;\mathrm{rate}\;\left(\%\right)=\left(\frac{\mathrm{The}\;\mathrm{number}\;\mathrm{of}\;\mathrm{colonies}\;\mathrm{after}\;\mathrm{drug}\;\mathrm{treatment}}{\mathrm{The}\;\mathrm{number}\;\mathrm{of}\;\mathrm{colonies}\;\mathrm{before}\;\mathrm{drug}\;\mathrm{treatment}}\right)\times 100$$

### 2.12. Preparation of Tumor Spheroids Using SKOV3-Red-Fluc and In Vitro 3D Tumor Spheroid Viability Assay

Tumor spheroids target cellularization with CSC properties [51,52]. SKOV3-red-fluc tumor spheroids were prepared according to previous studies [53]. Tumor spheroids were prepared using Matrigel (Corning, New York, NY, USA) as an extracellular matrix (ECM) [54,55]. Briefly, 10 μL was spherically dispensed into an ultra-low-attachment (ULA) 24-well plate, and 3 μL (1.0 × 10^4^ cells/μL) cell suspension was dispensed onto Matrigel. In an incubator at 37 °C, 2 mL/well of RPMI1640 was added per well. After 24 h, incubation was performed on an NB-101SRC orbital stirrer (N-BIOTEK, Bucheon, Republic of Korea). The medium was changed every 7 days. The morphology of the tumor spheroids was observed under a microscope on days 1, 10, and 22. Cytotoxicity analysis was performed 4 weeks after the preparation of tumor spheroids. IVIS images were obtained after treatment with d-luciferin prior to drug treatment. D-luciferin solution was prepared by dissolving a 200× stock solution in DPBS at a concentration of 30 mg/mL. After dilution to 150 µg/mL, 100 µL was dispensed into a 96-black well plate, the prepared tumor spheroid was added, and the luminescence intensity was measured after 10 min. In a 24-well plate, PTX solution, PTX micelles, PTX/SRF solution, and PTX/SRF micelles were treated at 62.8 µM based on PTX concentration. After 4 h, the cells were transferred to a ULA 24-well plate containing new media, placed on an orbital shaker (NB-1015RC), and cultured while stirring at 50 rpm. Luminescence images were obtained prior to and during the entire experimental period. Drugs were administered on days 4, 7, 11, and 14 based on the first drug treatment on day 0.

### 2.13. In Vivo Pharmacokinetic Evaluation and Biological Sample Preparation for HPLC Analysis

All animal experiments were conducted with the approval of the Animal Experiment Ethics Committee of Chungbuk National University (approval number: CBNUA1709-22-01). For pharmacokinetic evaluation, 7-week-old female Sprague-Dawley rats were purchased and evaluated after a stabilization period of 7 days. Solutions were prepared by dissolving PTX and SRF in EtOH: Cremophor EL^®^: sterile distilled water = 1:1:1 (*v*/*v*/*v*). PTX solution, PTX micelles, SRF solution, SRF micelles, PTX/SRF solution, and PTX/SRF micelles at a dose of 10 mg/kg were injected into the tail vein. At 5, 15, 30, 60, 120, 240, and 480 min after intravenous (IV) injection, the orbital plexus was ruptured using a heparin tube, and blood was collected. After the pretreatment of the blood sample, the concentration of the drug in the plasma was quantified by HPLC. PTX and SRF pharmacokinetic parameters and graphs were calculated using SigmaPlot 10.0 (Systat Software, Inc., San Jose, CA, USA). A 200 μL plasma supernatant sample, 400 μL of methanol, and 20 μL of IS were mixed, centrifuged at 4 °C for 5 min, and only the supernatant was filtered at 200 nm. Then, 200 μL of sample was added to the vial, of which 10 μL was injected into the HPLC system.

### 2.14. In Vivo Toxicity Assessment

Toxicity evaluation was performed after a stabilization period in a laboratory environment for at least 1 week after receiving 5-week-old ICR female mice. A formulation is considered toxic when >20% weight loss, abnormal behavior, discomfort, or death occur [56,57]. The control group was a non-treated group. The administration group was divided into a drug administration group and a DPBS administration group. Toxicity of formulation was assessed by concentration (10, 20, and 30 mg/kg) in an injected mouse tail vein. A solution was prepared by dissolving PTX and SRF using a solubilizer in the following volume ratio (EtOH: Cremophor EL^®^: sterile distilled water = 1:1:1, *v*/*v*/*v*). The micelles were prepared by hydration with sterile distilled water immediately before production using the freeze-drying method. Before administration, the exact concentration of the formulation was obtained using HPLC, and the injection volume was calculated to reflect the weight of each mouse and injected. The body weight of mice was measured on days 0 (first administration day), 2, 5, 8, 10, 13, 16, 18, 21, 24, 26, and 29, and the drug was injected every 8 days.

### 2.15. Preparation of Xenograft Nude Mice Model Using SKOV3-Red-Fluc and Luminescence Imaging

Female 6-week-old BALB/c nude mice (Orient Bio, Seongnam, Republic of Korea) were purchased and stabilized for 1 week prior to experimentation. To prepare the xenograft model, the SKOV3-red-fluc cell pellet was resuspended in DPBS, and Matrigel and cell suspension (containing 1 × 10^6^ cells) were subcutaneously injected using a 1 mL syringe (1:2, *v*/*v*). After growing the tumors for 3 weeks, the mice were randomly divided into five groups and subjected to antitumor evaluation. To evaluate the tumor growth inhibition (TGI, %) of PTX solution, PTX micelles, PTX/SRF solution, and PTX/SRF micelles, in vivo bioluminescent imaging (BLI) of tumors was performed before and after drug administration [58,59]. D-luciferin salt was prepared at a concentration of 15 mg/mL in DPBS and injected intraperitoneally at a dose of 150 mg/kg. Ten minutes after D-luciferin injection, fluorescence images were acquired with an in vivo optical imager (IVIS; VISQUE In Vivo Smart-LF, Republic of Korea), and luminescence signal intensity was measured and quantified using CleVue™ software version 3.1.3 2054 (Vieworks, Anyang, Republic of Korea).

$$\mathrm{Tumor}\;\mathrm{Growth}\;\mathrm{Inhibiton}\;\left(\mathrm{TGI},\;\%\right)=\left[1-\left(\frac{\mathrm{Experimental}\;\mathrm{group}\;\mathrm{Mean}\;\mathrm{BLI}}{\;\mathrm{Control}\;\mathrm{Mean}\;\mathrm{BLI}}\right)\right]\times 100\;$$

### 2.16. Antitumor Evaluation in Xenograft Nude Mice and H&E Staining

Five weeks after cancer cell transplantation, drugs were administered to the five groups. Mice were randomly relocated before the drug administration. The control group was intravenously injected with DPBS, and the experimental group was intravenously injected with PTX solution, PTX/SRF solution, PTX micelles, or PTX/SRF micelles (n = 5 for each group). The PTX dose in the experimental group was 20 mg/kg. The final dosing schedule was 0, 4, 8, 12, and 16 days, with a total of five injections. To evaluate toxicity while the antitumor evaluation was in progress, the first administration day was regarded as day 0, and body weight was measured every 2 days. At the end of the experiment, the tumors were collected after ethical euthanasia in a CO_2_ chamber. After collecting the tumor for H&E staining, slides were prepared according to the previous research method [60,61]. The slide is slide scanner Pannoramic SCAN II (3DHISTECH, Budapest, Hungary) scanning the cross section and obtained an image of CaseViewer 2.4 (3DHISTECH, Budapest, Hungary).

### 2.17. Statistical Analysis

All experiments used in the study were performed more than three times, and the resulting data are expressed as mean ± standard deviation (SD). Statistical significance was determined using a *t*-test in GraphPad Prism v 8.0 (GraphPad Software, La Jolla, CA, USA) and considered statistically significant when * *p* < 0.05, ** *p* < 0.01.

## 3. Results

### 3.1. Evaluation of the Synergistic Effect of PTX and SRF in OC Cell Lines

OC cell lines (HeyA8 and SKOV3-red-fluc) were treated with PTX and SRF at various molar ratios to obtain IC_50_ values, based on which, CI values were calculated. In Table 1, PTX:SRF = 1:2.3 (weight ratio PTX:SRF = 3 mg:3 mg), which shows a synergistic effect on both cell lines used in the experiment, was selected as the weight ratio of the final formulation.

### 3.2. Evaluation of Physicochemical Properties of Micelles Encapsulated with PTX and SRF

**Figure 2:**
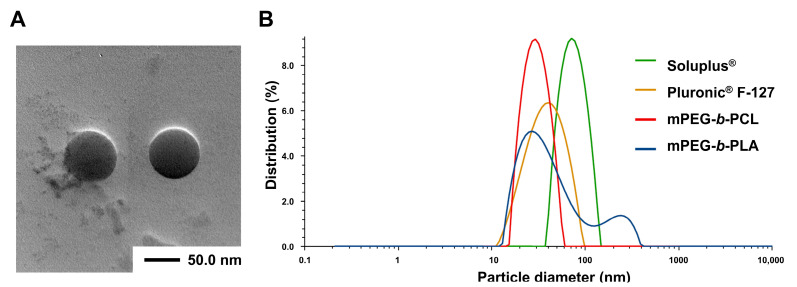
(**A**) Transmission electron microscopy (TEM) image of PTX/SRF-encapsulated mPEG-*b*-PCL micelles. (**B**) Representative particle size distributions of PTX/SRF-encapsulated micelles prepared with various polymers (PTX:SRF:polymer =3:3:100, weigh ratio, n = 3).

Physicochemical properties were evaluated to determine the optimal PTX/SRF-encapsulated micelles. Micelles encapsulated with a molar ratio combination (PTX:SRF = 1:2.3, 3:3 weight ratio) having a synergistic effect (CI < 0.5) on OC cell lines using various polymers (mPEG-*b*-PLA, F-127, Soluplus, mPEG-*b*-PCL) were prepared. Table 2 shows the EE (%), DL (%), particle size, PDI, and zeta potential of the prepared micelles. As a result of the physicochemical evaluation, mPEG-*b*-PCL, a polymer with the highest encapsulation efficiency (92.1 ± 7.76) and an ideal distribution in the size graph (Figure 2B), was selected as the final polymer. Subsequently, a physicochemical evaluation was performed by varying the amount of polymer to 60, 100, and 120 mg. The combination with the highest encapsulation efficiency (PTX 94.4 ± 4.14, SRF 91.6 ± 5.30), lowest particle size (33.1 ± 2.15 nm), PDI (0.23 ± 0.03), and weak negative charge (−0.12 ± 0.18) (PTX:SRF:mPEG-*b*-PCL = 3:3:60, weight ratio) was selected as the weight ratio of the final formulation. In Figure 2A, a TEM image of the final formulation shows spherical micelles smaller than 50 nm.

### 3.3. Stability Test

**Figure 3:**
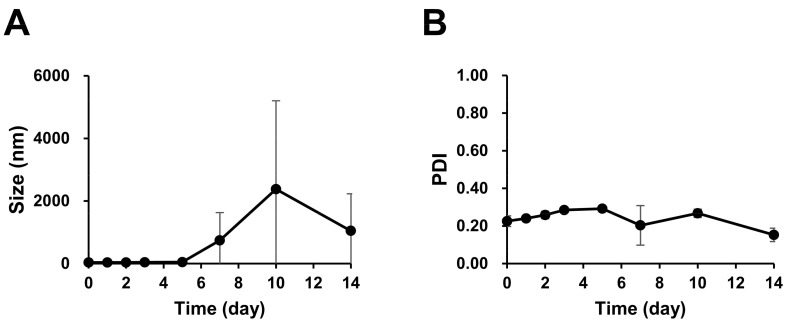
(**A**) Size and (**B**) PDI of PTX/SRF-encapsulated mPEG-*b*-PCL micelles stored at 4 °C for 2 weeks.

The size and PDI of PTX/SRF-encapsulated mPEG-*b*-PCL micelles were measured during 2 weeks of hydration at 4 °C refrigerated storage temperature. When stored at 4 °C, the average micelle size was less than 50 nm on day 5 but increased to ≤800 nm on day 7 (Figure 3A). The average PDI range of the final formulation was 0.2–0.3 (Figure 3B).

### 3.4. In Vitro Cytotoxicity Assay

**Figure 4:**
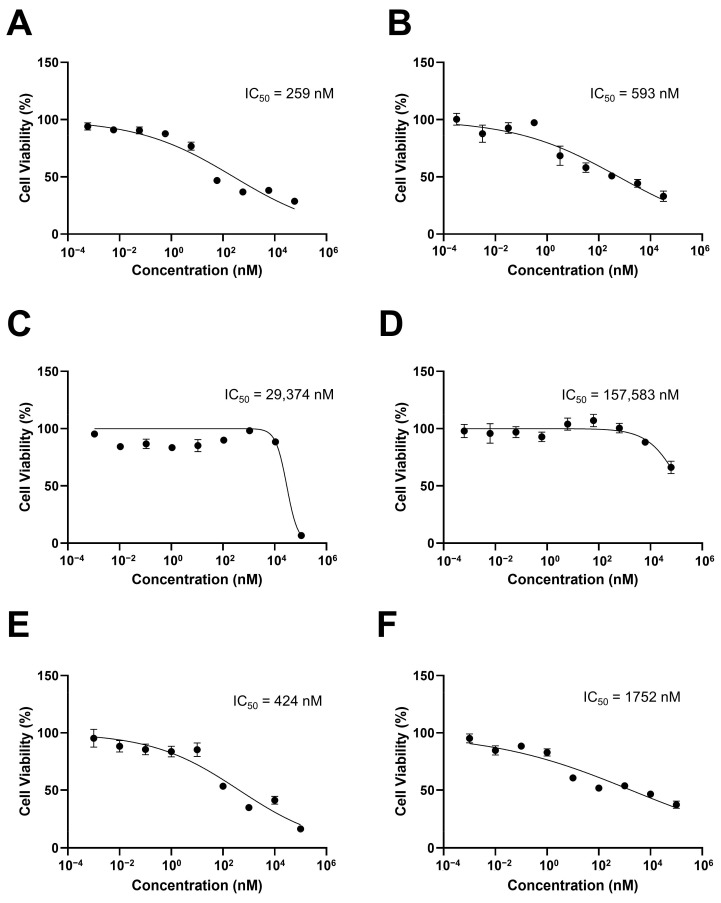
In vitro cytotoxicity analysis of SKOV3-red-fluc (**A**) PTX solution, (**B**) PTX micelle, (**C**) SRF solution, (**D**) SRF micelle, (**E**) PTX/SRF solution, and (**F**) PTX/SRF micelle (n = 6).

Figure 4 shows the results of the SKOV3-red-fluc cytotoxicity of free drugs and micelles when PTX and SRF were treated as a single drug or used in combination. The IC_50_ values of the free PTX, SRF, and PTX/SRF were 259, 29,374, and 424 nM, respectively. The IC_50_ values of PTX, SRF, and PTX/SRF micelles were 593, 157,583, and 1752 nM, respectively.

### 3.5. In Vitro Clonogenic Assay

**Figure 5:**
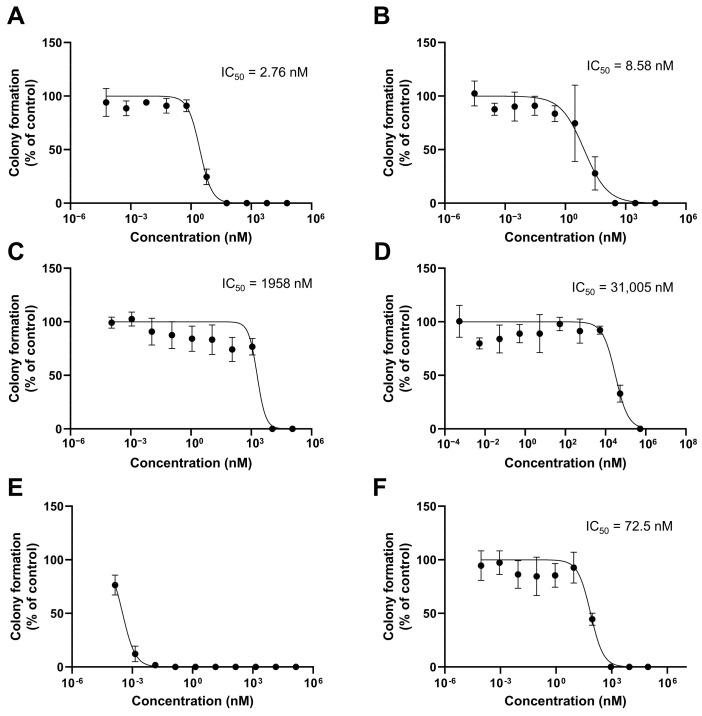
Colony formation inhibition in SKOV3-red-fluc cell line solution and micelles (**A**) free PTX, (**B**) PTX micelle, (**C**) free SRF, (**D**) SRF micelle, (**E**) free PTX/SRF, and (**F**) PTX/SRF micelle (n = 3).

After 2 weeks of treatment with PTX and SRF, colony formation inhibition was assessed (Figure 5). The IC_50_ values of the free PTX and SRF were 2.76 and 1958 nM, respectively. The IC_50_ value of the free PTX/SRF was not measured (Figure 5E). The IC_50_ values of PTX SRF, and PTX/SRF micelles were 8.58, 31,005, and 72.5 nM, respectively.

### 3.6. In Vitro Drug Release Profile

**Figure 6:**
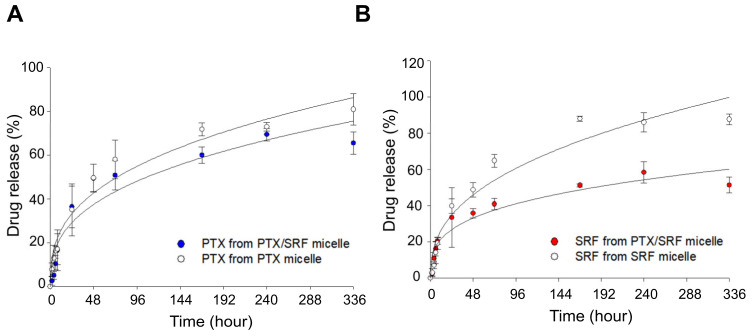
Drug release profiles of single micelles and combined micelles in vitro at 37 °C, pH 7.4. (**A**) PTX release pattern from PTX micelle and PTX/SRF micelle, (**B**) SRF release pattern from SRF micelle and PTX/SRF micelle (n = 3).

In the release profile, the PTX/SRF micelle combination released the drug at a slower rate than for individual micelles. As shown in Figure 6, PTX/SRF micelles and PTX micelles released 50.9% and 58.1% of PTX at 72 h, and 65.6% and 80.9% of PTX at 336 h, respectively. Further, SRF from PTX/SRF micelles and SRF micelles released 40.9% and 64.9% at 72 h, and 51.4% and 87.7% of SRF at 336 h, respectively.

### 3.7. In Vitro 3D Tumor Spheroid Viability Assay

**Figure 7:**
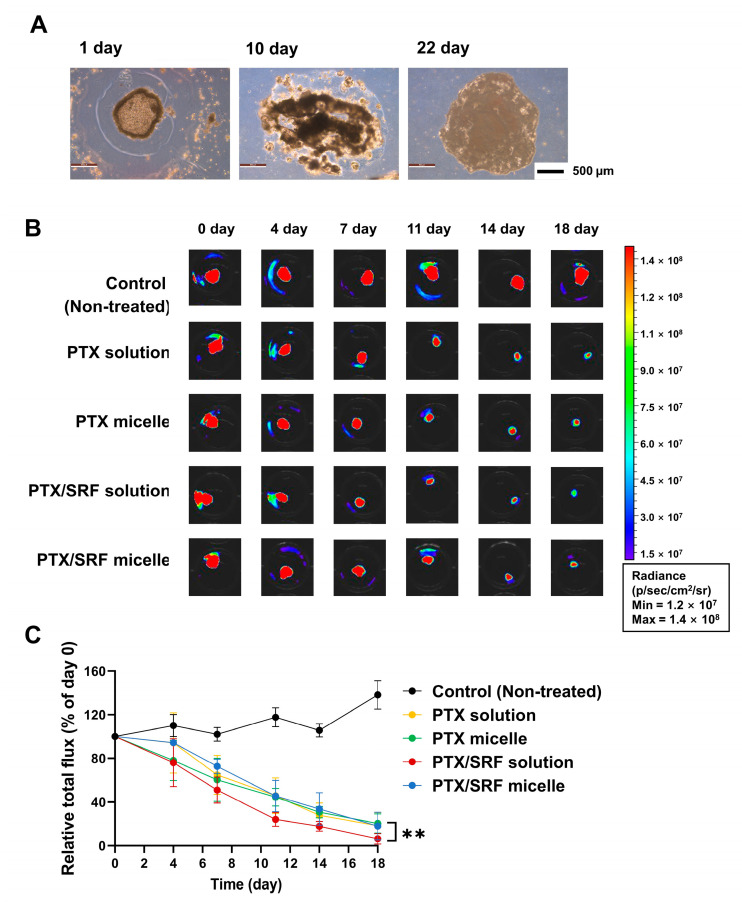
(**A**) Morphological observation of tumor spheroids prepared with SKOV3-red-fluc, (**B**) Control, PTX Solution, PTX micelles, PTX/SRF Solution, and PTX/SRF micelles SKOV3-red-fluc tumor spheroid IVIS measured with D-luciferin before treatment and after secondary treatment image (n = 4). (**C**) A graph showing the total luminescence value reflecting the tumor size for the first and second drug treatments based on the tumor spheroid before drug treatment (** *p <* 0.01, n = 4).

Tumor spheroids were observed microscopically on days 1, 10, and 22 after preparation (Figure 7A). After 24 h of tumor spheroid preparation, cell aggregation was observed inside the hemispherical matrigel. Tumor spheres were then detached from the bottom of the ULA plate, and the cells were aggregated and grown on matrigel cores. After 22 days, it was found to be three-dimensionally close to a sphere. Compared with day 0, the total relative luminescence intensity during the entire experimental period increased in the control group but decreased in the drug-treated group (Figure 7B). Compared to the control group, the average total flux value decreased by 85.5%, 84.9%, 94.7%, and 85.9% in the order of PTX solution, PTX micelles, PTX/SRF solution, and PTX/SRF micelles, respectively (Figure 7C). At the end of the experiment, there was a significant difference in the relative total flux between the PTX solution and PTX micelles (** *p* < 0.01), and between the PTX/SRF solution and PTX/SRF micelles (** *p* < 0.01).

### 3.8. Pharmacokinetic Evaluation

**Figure 8:**
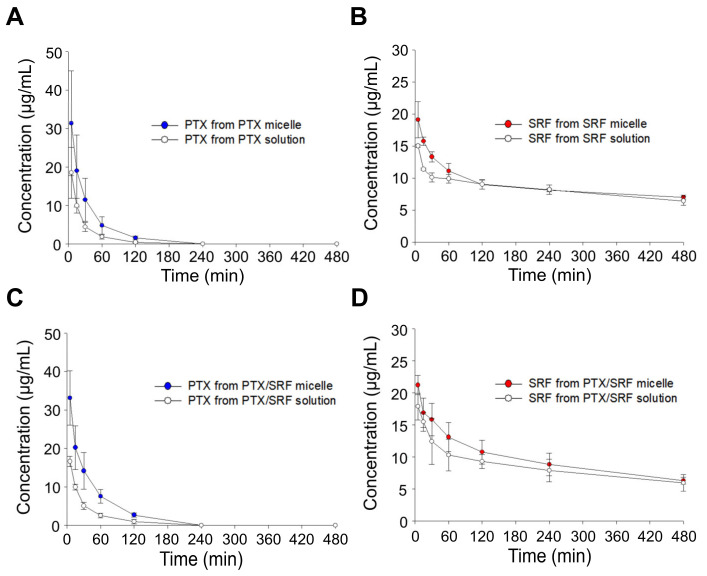
Plasma concentration–time profiles of PTX and SRF after IV injection in SD female rats, (**A**) PTX from PTX solution and PTX micelle, (**B**) SRF from SRF solution and SRF micelle, (**C**) PTX from PTX/SRF solution and PTX/SRF micelle, (**D**) SRF from PTX/SRF solution and PTX/SRF micelle (n = 3).

In Figure 8, the pharmacokinetic evaluation of 7-week-old female SD rats revealed that PTX was detected below the quantification limit in plasma 4 h after IV injection, and SRF was detected even 8 h after injection. In all combinations, the bioavailability of micelles increased compared to that of the solution, and the combination of PTX/SRF micelle showed higher bioavailability than the single drug solution and micelles (Table 3 and Table 4).

### 3.9. In Vivo Toxicity Assessment

**Figure 9:**
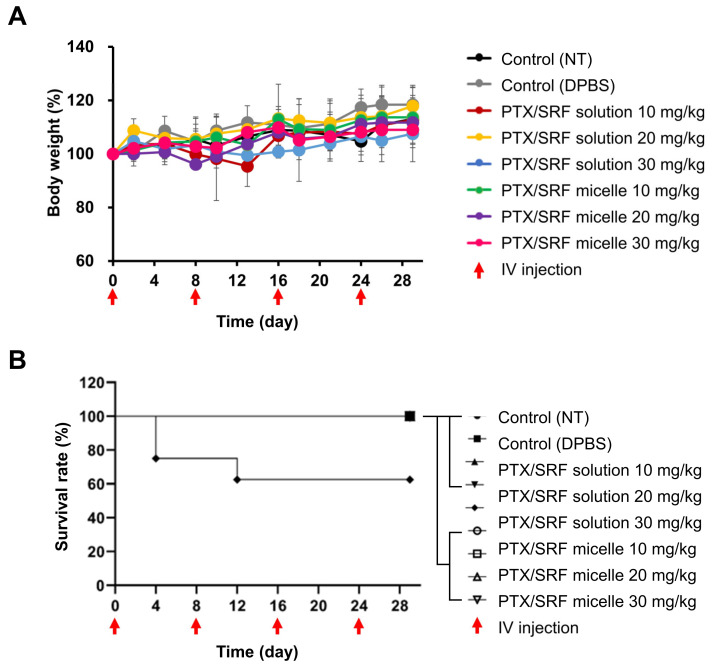
(**A**) Weight analysis at day 0 for in vivo toxicity evaluation of various concentrations of solution and micelle. (**B**) Kaplan–Meier graph showing toxicity assessment survival rate (n = 5).

The ICR mouse toxicity evaluation revealed that neither the solution nor the micelles caused toxicity that resulted in a change of 20% of the pre-administration weight (Figure 9A). A 30 mg/kg solution weakened the breathing of mice immediately after administration, and 20% of the mice were sacrificed (Figure 9B). In the second administration, 25% of the mice that survived after the first administration were sacrificed. In contrast, in the group administered micelles hydrated with sterile distilled water, IV injection into the tail vein of ICR mice showed no signs of toxicity during the entire experimental period.

### 3.10. Antitumor Evaluation and H&E Staining and Toxicity Assessment

**Figure 10:**
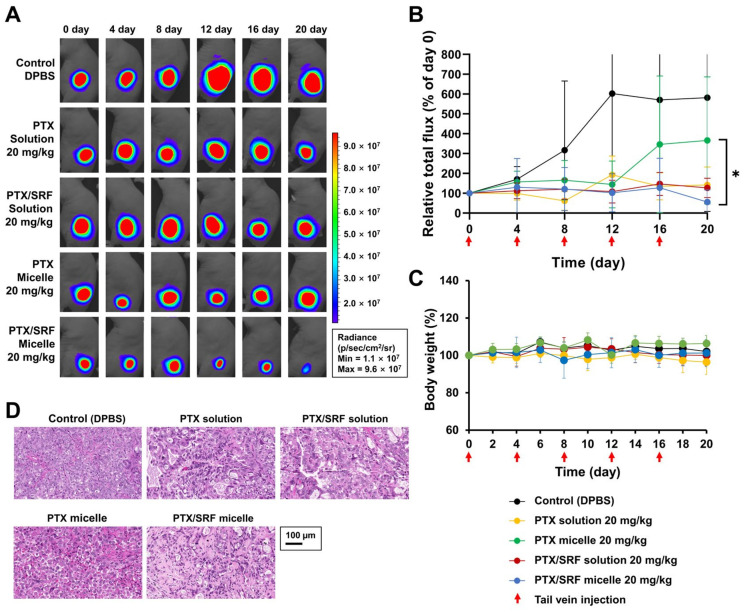
(**A**) Representative IVIS images of an SKOV3-red-fluc cell xenograft model taken 20 days after IV injection with DPBS (control), solution, and micelle, (**B**) relative total flux (% of day 9) value graph of each group (* *p <* 0.05, n = 5), (**C**) body weight change, (**D**) representative H&E staining of xenograft mouse model.

In a xenograft nude mouse model of OC, the TGI (%) was 75.6, 78.2, 37.0, and 90.4, respectively (Figure 10B). DPBS was used as a control in all formulations. There was a significant difference compared with the treatment group (* *p* < 0.05). In the case of the solution, there was no difference between single and combined use, whereas the results showed a significant difference between micelles (* *p* < 0.05). The antitumor evaluation using xenograft nude mice demonstrated that the PTX/SRF micelle group showed the highest tumor growth inhibition rate (99.4%). Xenograft female mice bearing SKOV3-red-fluc showed no 10% reduction weight loss during the entire antitumor evaluation period (Figure 10C). There was no significant difference in weight change between the PTX micelle and PTX/SRF micelle formulation administration groups compared to the DPBS administration group. The PTX/SRF solution administration group showed a difference from the PTX solution administration group (* *p* < 0.05). Upon obtaining tumors after the antitumor evaluation was complete, H&E staining confirmed that the cells of the DPBS injection group were dense, their morphology was intact, and their compartmentalization was intact. New blood vessels were observed around the mass in the PTX single administration group. Figure 10D shows that the PTX/SRF combination group shows the most distinct degree of apoptosis.

## 4. Discussion

Most OCs are epithelial, and the majority are serous carcinomas. It is difficult to diagnose OC at an early stage; therefore, it is likely to be detected at stage three or higher [3,62]. Although surgical and chemotherapy strategies for treating OC are evolving [63], the 5-year overall survival rate for OC remains low at 40%. The development of novel treatments for OC is, therefore, needed; micelles, used in combination therapy, may be an alternative [64,65]. The advantage of combination therapy is that anticancer drugs with different mechanisms can be applied to OC treatment. They can suppress cancer more effectively than individual agents. [66] PTX, a taxane-based anticancer agent, is used as the standard treatment for OC [67]. SRF, a TKI drug that inhibits angiogenesis, has been applied to the treatment of OC [20,29]. Combination therapy with drugs with different mechanisms, such as PTX and sorafenib (SRF), can achieve a synergistic effect; therefore, improved anticancer effects can be expected compared to monotherapy [66].

MTT assay was performed to determine the molar ratio between the synergistic effect of PTX and SRF, drugs with different anticancer mechanisms, in OC cell lines. A molar ratio (PTX:SRF = 1:2.3) showing a synergistic effect in the two OC cell lines (SKOV3-red-fluc and HeyA8) was found. According to previous pharmacokinetic analysis studies, it has been reported that SRF remains in vivo longer than PTX [68]. Based on this, we selected 1:2.3, which was the combination of the lowest molar ratio of SRF among several molar ratios with a synergistic effect, as the final molar ratio.

We prepared micellar formulations by freeze-drying four types of polymers. When micelles are produced via the freeze-drying method, storage is convenient as they can be stored frozen in powder form. The physical properties of micelles prepared from four different polymers encapsulated with PTX/SRF were analyzed. We used pre-warmed solvent to increase EE after freeze-drying the final formulation, followed by a sonication process. This may act as a limitation when applied to clinical practice in the future. It is necessary to devise a simpler method while maintaining a higher EE through future research. We excluded mPEG-*b*-PLA from the final candidates due to the non-normal particle size distribution by DLS, and Soluplus^®^ was excluded due to low EE (%). mPEG-*b*-PCL, which was selected as the final polymer, had the advantages of the highest EE (%), low PDI, and ideal size. Subsequently, when the optimization process was performed by varying the amount of mPEG-*b*-PCL polymer, the EE (%) and DL (%) tended to decrease as the polymer weight increased, the particle size increased, and the absolute value of the zeta potential increased. The particle size of the final micelles (PTX:SRF:mPEG-*b*-PCL=3:3:60, mg) evaluated by DLS was <50 nm, the PDI value was 0.3, indicating a monodisperse particle distribution, and the zeta potential was weakly negative. It has the characteristic of accumulating steadily in tumors in vivo [69]. As a result of the stability evaluation, PTX/SRF micelles were found to maintain stability for up to 5 days when stored at 4 °C. Amphiphilic mPEG-*b*-PCL, the polymer of the final formulation, has the advantage of low critical micelle concentration when producing micelles and stability during hydration compared to other charged polymers [70].

In the MTT assay performed on the SKOV3-red-fluc cell line to evaluate cytotoxicity, the free drug showed a lower IC_50_ value than the drug encapsulated in micelles. The CI value of free PTX/SRF was 0.501, which indicated a synergistic effect, while the CI of PTX/SRF micelles was 0.894, which was CI < 1, indicating that a synergistic effect was still observed. For micelles, it can be hypothesized that the PTX/SRF from micelles was released at a slower rate than the free drug. This is because it was absorbed into cancer cells, increasing the CI value [65,71]. In the in vitro cytotoxicity evaluation, the free drug had a lower IC_50_ value than did the micelle formulation as it is applied directly to cancer cells. In contrast, micelles are absorbed and released by cancer cells through the release of encapsulated PTX and SRF. As a result of the time required for the endocytosis of micelles into cancer cells, the IC_50_ value may be higher than that of the free drug. This results in a higher CI value. PTX micelles, SRF micelles, and PTX/SRF micelles showed lower IC_50_ values in the clonogenic assay than free PTX, free SRF, and free PTX/SRF, which showed the same tendency as the MTT assay.

We prepared tumor spheroids to evaluate cytotoxicity in a three-dimensional environment [72,73]. The purpose of this assay was to investigate the effect of PTX/SRF micelles on multi-layered cancer cells rather than on single-layered cancer cells. As a result of the sixth drug treatment, the average total flux value of free PTX/SRF was greatly reduced. This indicates that free PTX/SRF exhibits stronger anticancer activity than micelle PTX/SRF, as demonstrated by MTT and clonogenic analysis. These results can be attributed to the difficulty in reflecting the anticancer effect of mechanisms, such as the inhibition of angiogenesis by SRF in an in vitro environment.

In vivo toxicity experiments using ICR mice showed that tail vein inflammation, edema, and necrosis occurred immediately after the administration of a free drug using a solubilizing agent. This is believed to be due to the toxicity of Cremophor EL^®^, a solubilizing agent [74]. In contrast, in the in vivo toxicity evaluation of the final formulation, the PTX micelle and PTX/SRF micelle formulations showed no significant difference in weight gain when compared with the DPBS-administered group. This indicates that the micelle formulation has lower toxicity than the solution formulation [75].

In vitro release experiments revealed that PTX and SRF derived from the PTX/SRF micelle formulation released more slowly than each single drug micelle. This is speculated to be due to the hydrophobic attraction between poorly soluble drugs within the micellar core [76]. Considering the sink conditions, despite the use of 2L of PBS, the drug was not released 100% due to the following two reasons. (1) In in vitro release experiments, it was observed that the polymer was entangled in the semi-permeable membrane, which may have resulted in less than 100% drug release. (2) This may be due to the specific interaction of sorafenib with the hydrophilic side chain of the block copolymer [77]. In the pharmacokinetic evaluation, single micelles and combined micelles showed improved bioavailability and a lower CLt than each solution formulation. Due to its size of larger than 20 nm, the mPEG-*b*-PCL micelle formulation was not removed by RES, and as it was smaller than 100 nm, it was possible to avoid macrophage phagocytosis [78]. In conclusion, the micellar formulation can remain in vivo for an extended period owing to the EPR effect [20,36,37]. In addition, in the case of PTX/SRF-combined micelles, pharmacokinetic parameters were better than those of single micelles, which is consistent with in vitro release results.

In the evaluation of the anticancer effect in the xenograft nude mouse tumor model, the PTX/SRF-micelle-administered group showed the highest tumor growth inhibition rate (99.4%) at the end of the experiment. This is a tumor growth inhibitory effect that is 2.45-times more effective than the PTX-micelle-administered group. Consequently, this suggests that the anticancer effect of PTX is enhanced compared to that of PTX alone in treating OC. A PTX/SRF micelle formulation, unlike in vitro, improved the anticancer effect of free PTX/SRF in vivo. This is due to the angiogenesis inhibitory effect, which is the mechanism of action of SRF. During the process of recovering the subcutaneously transplanted tumor following the antitumor evaluation, it was confirmed that angiogenesis to the tumor was inhibited in the SRF/PTX combined experimental group. H&E analysis of the collected tumors confirmed that the cells in the DPBS-injected group (control group) were dense, intact, and compartmentalized. Several sites have been observed where chromosomes condense during mitosis. Conversely, for the drug-administered group, it was observed that apoptosis occurred not only in the center, where the boundary of cancer cells was not intact, but also in the periphery. H&E staining showed that PTX/SRF micelles had a stronger anticancer effect than the control and PTX micelles. In conclusion, mPEG-*b*-PCL micelles combined with PTX and SRF for OC treatment are synergistic combinations. In addition, in vivo experiments showed that the anticancer effect was improved compared with existing solution formulations or single-drug therapy. Previous research dealt with anticancer evaluation of PTX/SRF-encapsulated nanoparticles evaluated in liver and breast cancer [42,43]. This study is valuable as the first PTX/SRF combined micelle study evaluated in OC. The results of this study demonstrate the potential of PTX/SRF micelles as an injectable drug for the treatment of OC, which may provide a novel approach to its treatment.

## 5. Conclusions

Combination therapy can yield a synergistic effect in the treatment of OC compared to monotherapy. Free PTX and free SRF had a synergistic effect (CI < 0.5) on the OC cell lines (HeyA8 and SKOV3-red-fluc) at a molar ratio of 1:2.3. The final formulation, mPEG-*b*-PCL micelles encapsulated with PTX and SRF, exhibited high EE (%), low PDI, and weak negative charge and maintained stability at 4 °C for up to 5 days after hydration. Based on the in vitro evaluation of cytotoxicity in tumor spheroids prepared with SKOV3 cells, the PTX/SRF solution showed the highest rate of tumor spheroid growth inhibition. A similar trend has been observed in the conventional MTT results, and it is considered that the in vitro evaluation does not reflect the angiogenesis inhibitory effect of SRF. Considering the excellent tumor suppression effect of the final formulation, additional studies in a preclinical environment are required for the clinical evaluation of PTX/SRF micelle combination therapy for the treatment of OC. 

## Figures and Tables

**Table 1:** IC_50_ and CI values of paclitaxel (PTX) and sorafenib (SRF) at various molar ratios (n = 3).

PTX:SRF (Molar Ratio)	SKOV3-Red-Fluc			HeyA8		
	IC_50_ (nM)		CI Value	IC_50_ (nM)		CI Value
	PTX	SRF		PTX	SRF	
1:1	226	226	0.882	109	109	0.447
1:1.5	131	196	0.511	60.6	91.0	0.255
1:2.3	127	297	0.501	75.8	177	0.331
1:4	107	427	0.427	71.0	284	0.332
1:9	105	946	0.438	67.3	606	0.377

**Table 2:** Physicochemical properties of formulations using various concentrations of mixed various polymers (n = 3).

Polymer Category	Amount of Polymer Used (mg)	Amount of PTX Used (mg)	Amount of SRF Used (mg)	PTX Encapsulation Efficiency (EE, %)	SRF Encapsulation Efficiency (EE, %)	PTX Drug Loading (DL, %)	SRF Drug Loading (DL, %)	Particle Size (nm)	PolyDispersity Index (PDI)	Zeta Potential (mV)
mPEG-*b*-PLA	100	3	3	83.0 ± 4.83	84.6 ± 5.90	2.35 ± 0.16	2.48 ± 0.13	65.3 ± 12.3	0.35 ± 0.01	−9.90 ± 8.31
F-127	100	3	3	81.5 ± 1.45	81.0 ± 0.99	2.31 ± 0.03	2.38 ± 0.15	39.9 ± 7.43	0.28 ± 0.04	−5.58 ± 5.83
Soluplus^®^	100	3	3	14.6 ± 9.22	15.7 ± 11.1	0.41 ± 0.31	0.45 ± 0.25	76.1 ± 10.1	0.12 ± 0.04	−3.92 ± 3.83
mPEG-*b*-PCL	60	3	3	94.4 ± 4.14	91.6 ± 5.30	4.29 ± 0.18	4.17 ± 0.24	33.1 ± 2.15	0.23 ± 0.03	−0.12 ± 0.18
	100	3	3	92.1 ± 7.76	90.2 ± 9.21	2.61 ± 0.26	2.64 ± 0.22	39.2 ± 1.15	0.28 ± 0.02	−8.42 ± 2.93
	120	3	3	87.6 ± 6.46	86.1 ± 4.06	2.09 ± 0.15	2.05 ± 0.09	40.3 ± 4.36	0.26 ± 0.02	−11.3 ± 2.74

**Table 3:** Pharmacokinetic parameters of PTX after intravenous (IV) injection of PTX solution, PTX mPEG-*b*-PCL micelles, PTX/SRF solution, and PTX/SRF-mPEG-*b*-PCL micelles in rats (n = 3).

Parameter	PTX Solution	PTX Micelle	PTX in PTX/SRF Solution	PTX In PTX/SRF Micelle
Dose (µg∙kg^−1^)	1000	1000	1000	1000
AUC_0–8h_ (µg∙min∙mL^−1^)	440 ± 237	1020 ± 628	531 ± 281	1320 ± 712
CLt (mL∙(kg∙min)^−1^)	23.8 ± 13.0	11.2 ± 6.79	19.4 ± 10.3	7.90 ± 4.34
Relative bioavailability	-	232	-	249

**Table 4:** Pharmacokinetic parameters of SRF after IV injection of SRF-solution, SRF-mPEG-*b*-PCL micelles, PTX/SRF solution, and PTX/SRF-mPEG-*b*-PCL micelles in rats (n = 3).

Parameter	SRF Solution	SRF Micelle	SRF in PTX/SRF Solution	SRF in PTX/SRF Micelle
Dose (µg∙kg^−1^)	1000	1000	1000	1000
AUC_0–8h_ (µg∙min∙mL^−1^)	3960 ± 200	4210 ± 2110	3720 ± 1880	4580 ± 2360
CLt (mL∙(kg∙min)^−1^)	2.54 ± 1.28	2.38 ± 1.19	2.70 ± 1.36	2.22 ± 1.14
Relative bioavailability	-	106	-	123

Abbreviations: AUC; area under the curve from 0–8 h, CL_t_; total clearance.

## Data Availability

The data presented in this article are contained in the manuscript.
